# Psychological Distress, Depression, Anxiety, and Burnout among International Humanitarian Aid Workers: A Longitudinal Study

**DOI:** 10.1371/journal.pone.0044948

**Published:** 2012-09-12

**Authors:** Barbara Lopes Cardozo, Carol Gotway Crawford, Cynthia Eriksson, Julia Zhu, Miriam Sabin, Alastair Ager, David Foy, Leslie Snider, Willem Scholte, Reinhard Kaiser, Miranda Olff, Bas Rijnen, Winnifred Simon

**Affiliations:** 1 Center for Global Health, International Emergency and Refugee Health Branch, Centers for Disease Control and Prevention (CDC), Atlanta, Georgia, United States of America; 2 Office of Surveillance, Epidemiology, and Laboratory Services, Centers for Disease Control and Prevention (CDC), Atlanta, Georgia, United States of America; 3 Academic Medical Center, University of Amsterdam, Amsterdam, The Netherlands; 4 Pepperdine University, Malibu, California, United States of America; 5 Fuller Theological Seminary, Pasadena, California, United States of America; 6 Tulane University School of Public Health and Tropical Medicine, New Orleans, Louisiana, United States of America; 7 Antares Foundation, Amsterdam, The Netherlands; Wayne State University, United States of America

## Abstract

**Background:**

International humanitarian aid workers providing care in emergencies are subjected to numerous chronic and traumatic stressors.

**Objectives:**

To examine consequences of such experiences on aid workers' mental health and how the impact is influenced by moderating variables.

**Methodology:**

We conducted a longitudinal study in a sample of international non-governmental organizations. Study outcomes included anxiety, depression, burnout, and life and job satisfaction. We performed bivariate regression analyses at three time points. We fitted generalized estimating equation multivariable regression models for the longitudinal analyses.

**Results:**

Study participants from 19 NGOs were assessed at three time points: 212 participated at pre-deployment; 169 (80%) post-deployment; and 154 (73%) within 3–6 months after deployment. Prior to deployment, 12 (3.8%) participants reported anxiety symptoms, compared to 20 (11.8%) at post-deployment (p = 0·0027); 22 (10.4%) reported depression symptoms, compared to 33 (19.5%) at post-deployment (p = 0·0117) and 31 (20.1%) at follow-up (p = .00083). History of mental illness (adjusted odds ratio [AOR] 4.2; 95% confidence interval [CI] 1·45–12·50) contributed to an increased risk for anxiety. The experience of extraordinary stress was a contributor to increased risk for burnout depersonalization (AOR 1.5; 95% CI 1.17–1.83). Higher levels of chronic stress exposure during deployment were contributors to an increased risk for depression (AOR 1·1; 95% CI 1·02–1.20) comparing post- versus pre-deployment, and increased risk for burnout emotional exhaustion (AOR 1.1; 95% CI 1.04–1.19). Social support was associated with lower levels of depression (AOR 0·9; 95% CI 0·84–0·95), psychological distress (AOR = 0.9; [CI] 0.85–0.97), burnout lack of personal accomplishment (AOR 0·95; 95% CI 0·91–0·98), and greater life satisfaction (p = 0.0213).

**Conclusions:**

When recruiting and preparing aid workers for deployment, organizations should consider history of mental illness and take steps to decrease chronic stressors, and strengthen social support networks.

## Introduction

International humanitarian aid workers are increasingly at high risk for experiencing violence [1] and being exposed to terrorism and direct attacks (e.g., Iraq and Afghanistan). Such extreme distress may result in negative mental health consequences, which in turn may affect the functioning and productivity of the aid organizations. Other stressors (e.g., job insecurity, restricted career development opportunities, low salaries, or unsafe living conditions) may also lead to burnout and other negative mental health outcomes [2]. Humanitarian aid organizations have begun to identify the need for an organizational policy and response to the psychological consequences of humanitarian work. Agencies and individuals have proposed programs oriented to select, train, and support staff [3]–[5]; however, a serious lack of scientific knowledge hampers organizations in managing and supporting staff and improving worker productivity [6]–[8].

This article describes, to the best of our knowledge, the first longitudinal study among expatriate humanitarian aid workers in a representative sample of non-governmental organizations (NGOs). We aimed to establish predictive associations between personal, organizational, and work-related stressors, and negative mental health outcomes, burnout, and life satisfaction. In this article, we focus on the longitudinal results of the data at pre-deployment, post-deployment and 3 to 6 months post-deployment.

### Hypotheses, study goals and objectives

We hypothesized that exposure to risk factors, such as exposure to trauma and chronic stressors, as well as protective factors, such as social support, healthy lifestyle and healthy coping strategies, would be significantly associated with mental health outcomes (depression, anxiety) and burnout. These hypotheses and expected changes were based on previous cross-sectional studies among humanitarian aid workers [2], [4], [5], [6], [7], [8]. A secondary focus of the study was to identify the prevalence of anxiety, depression, and burnout in this aid worker sample.

The goal of this study was to provide scientific evidence that work and job-related stressors are associated with mental distress and burnout, and risk and mitigating factors moderate the impact of such stressors among expatriate humanitarian aid workers. The objectives were to provide recommendations for selecting, training, and managing workers to reduce these stressors. We selected identifiable stressors during deployment (traumatic events or duty-related experience) that can predict negative mental health outcomes and examined the influence of identifiable moderating variables (prior trauma experience, social support, organizational culture, and living conditions).

**Table 1:** Demographic Characteristics of the Humanitarian Aid Worker Sample.

Variable	n/N (%) Pre-deployment	n/N (%) Post- deployment	n/N (%) Follow-up
SexMaleFemale	86/211 (40.8)125/211 (59.2)	––	––
Age	*M* = 34.2 (*SD* = 8.54) Range = 22 – 65	*M* = 34.5 (*SD* = 8.18)Range = 22 – 65	*M* = 34.8 (*SD* = 8.79)Range = 22 – 65
Marital StatusSingleMarriedIn Committed relationshipSeparated/Divorced/Widowed	113/212 (54.1)37/212 (17.5)49/212 (23.1)13/212 (6.1)	92/170 (54.1)28/170 (16.5)40/170 (23.5)10/170 (5.9)	85/154 (55.6)24/154 (15.7)35/154 (22.9)9/154 (5.9)
Educational LevelHigh school/VocationalUniversityPostgraduate	25/211 (11.9)140/211 (66.4)46/211 (21.8)	–––	–––
Job Function /current employment statusHead of Mission/ Regional DirectorManager/ CoordinatorTechnical Program staffLogistics StaffAdministrative StaffOther	2/212 (.9) 62/212 (29.2)72/212 (34.0)29/212 (13.7)21/212 (9.9)26/212(12.3)	6/170 (3.6)46/170 (27.2)56/170 (33.1)19/170 (11.2)15/170 (8.9)27/170 (16.0)	2/154 (1.3)30/154 (19.9)24/154 (15.9)9/154 (6.0)8/154 (5.3)78/154 (51.7)
Number of Previous Humanitarian Field AssignmentsNo prior assignments1 assignment2 – 4 assignments5 – 9 assignments>10 assignments	64/212(30.2)38/212(17.9)74/212(34.9)28/212(13.2)8/212(3.8)	–––––	–––––
Previous Mental Illness	41/212 (19.3)	–	–

## Methods

### Ethics Statement

The study protocol was reviewed and approved by the U.S. Department of Health and Human Services/Centers for Disease Control and Prevention (CDC) Institutional Review Board. All participating organizations and institutions deferred to CDC's IRB approval of the study protocol except Tulane University, which conducted its own ethics review and approved the protocol. We obtained written consent from all study participants.

### Study design and participation

Our longitudinal study included regular measurement intervals at pre-deployment (Time 1), post-deployment (Time 2), and 3–6 months post-deployment (Time 3). We established inclusion criteria for participating agencies as: being in existence for >5 years; having an established record of international funding; operating with a humanitarian imperative (emergency aid and development); a record of operations in countries at risk for widespread violence; including low-income countries or those affected by chronic crisis; deploying a minimum of 20 expatriate staff to the field/year. Minimum length of deployment for individual participant inclusion was 3 months and maximum length was 12 months.

To give the desired power and account for potential loss to follow up, we aimed to recruit 250 aid workers in total. To determine the required sample size, a relatively large correlation among survey responses over time (corr = 0.8) through time was assumed. Thus, assuming a true prevalence of 10% of mental health-related problems or problems in occupational-, or social functioning, a minimum sample size of 250 persons would be required if the risk ratio to be detected is 2, with a confidence level of 95%, and a power (Beta-1) of 80%.

**Table 2:** Association between stressors and risk/mitigating factors and depression and anxiety, and how these changes across time, compared with pre-deployment as baseline*.

	AOR post versus pre (95% CI)	AOR follow-up versus pre (95% CI)	Type III p value†
**Depression**			
Chronic stress*Sum	1·11 (1·02–1·20)	1·03 (0·93–1·14)	**0·005**
Trauma exposure category§			
Traumatic stress category 2 versus 1	1.57 (0.54–4.53)	0.31 (0.12–0.80)	**0·055**
Traumatic stress category 3 versus 1	1.80 (0.37–8.86)	0.78 (0.12– 5.11)	
Extraordinary stress	1.10 (0.81–1.47)	0.88 (0.64–1.19)	**0·041**
**Anxiety**			
Health index	0.91 (0.54–1.53)	0.57 (0.35–0.90)	0·116

AOR  =  adjusted odds ratio; CI  =  confidence interval.

p values <0·05 were considered statistically significant and are in bold type.

* Chronic stress and time are interacting, which means that the effects of chronic stress on depression are different among pre-deployment, post-deployment, and follow-up.

† p value: predictor × time interactions.

§ Trauma exposures are defined as follows:

Category 1 = 0 trauma events.

Category 2 = 1–4 traumatic events.

Category 3 = ≥5 traumatic events.

NGOs were recruited from a list of agencies meeting the inclusion criteria, in part based on the Relief Web archive (http://www.reliefweb.int). An initial list of 88 NGOs was compiled from descriptions available on the Relief Web archive (http://www.reliefweb.int). The researchers reached out to these 88 organizations to verify if they met the inclusion criteria and to determine if they were willing to participate. The size of the organizations was determined during this recruitment process. Out of 88 organizations, 18 did not respond to queries, 22 did not provide information to determine suitability for inclusion and eight were identified as not meeting inclusion criteria. Of the 40 agencies confirmed to meet inclusion criteria, 21 declined the invitation to participate, leaving 19 participating agencies.

**Table 3:** Longitudinal multivariate generalized estimating equations models: demographic variables, exposure, organizational and other risk and mitigating factors across time affecting anxiety, depression, and psychological distress.

	Anxiety		Depression		Psychological Distress	
Parameter	AOR (95% CI)	p value	AOR (95% CI)	p value	AOR (95% CI)	p value
**Sex**Male versus female	0·54 (0·19–1·53)	0·270	0·77 (0·35–1·72)	0·522	0.68 (0.30–1.51)	0.351
**Age**	0·95 (0·88–1·04)	0·222	0·98 (0·93–1·02)	0·278	0.99 (0.94–1.04)	0.769
**Marital status**Not married versus married	0·83 (0·31–2·27)	0·725	0·50 (0·26–0·98)	0·054	0.31 (0.15–0.65)	**0.005**
**Job function**Non-manager versus head of mission	0.63 (0·23–1.74)	0·414	0·58 (0·30–1·14)	0·127	0.89 (0.41–1.94)	0.769
**Hardship assignment**						
Yes versus no	1·86 (0·52–6·57)		0·42 (0·17–1·03)		0.33 (0.13–0.80)	
Agency does not designate any hardship assignments versus no	2·35 (0·45–12·32)	0·718	0·44 (0·18–1·03)	0·255	0.28 (0.11–0.74)	0.067
Don't know versus no	1·88 (0·41–8·53)		0·53 (0·17–1·68)		0.41 (0.13–1.34)	
**History mental illness** No versus yes	0·24 (0·08–0·69)	**0·016**	0·47 (0·22–1·01)	0·072	–	–
**NGO evaluation** sum	1·09 (1·01–1·19)	**0·033**	–	–	0.95 (0.87–1.04)	0.299
Trauma exposure category*						
Traumatic stress category 2 versus 1	0·46 (0·17–1·24)	0·101	–	–	–	–
Traumatic stress Category 3 versus 1	1·92 (0·53–6·90)		–	–	–	–
**Team cohesion** **field leader**	0·93 (0·84–1·03)	0·235	–	–	–	–
**Social support**	0·95 (0·89–1·02)	0·215	0·89 (0·84–0·95)	**0·0001**	0.91 (0.85–0.97)	**0.004**
**Motivation**	–	–	–	–	1.07 (0.98–1.17)	0.129
**Child trauma**	1·67 (0·86–3·24)	0·174	1·40 (0·89–2·21)	0·155	1.73 (0.78–3.85)	0.169
**Extraordinary** **stress**	1·01 (0·74–1·37)	0·952	–	–	0.99 (0.76–1.28)	0.943
**Health habits** **index**	–	–	0·89 (0·69–1·15	0·371	0.84 (0.66–1.06)	0.157
**Adult trauma**	–	–	1·33 (0·73–2·42)	0·347	2.71 (1.35–5.48)	**0.009**

AOR  =  adjusted odds ratio; CI  =  confidence interval.

Each variable in the table was adjusted for all other variables in the table.

Time was also included as an adjustment variable in the analysis.

p values are based on the Type III Wald chi-squared statistic.

p values <0·05 were considered statistically significant and are in bold type.

* Trauma exposures are defined as follows:

Category 1 = 0 trauma events.

Category 2 = 1–4 traumatic events.

Category 3 = ≥5 traumatic events.

The size of the organization was taken into consideration for worker recruitment, as the number of annual deployments across participating agencies ranged from 20 to 700. The research coordinator sent each agency contact person the appropriate number of pre-deployment questionnaires in proportion to its size. At the beginning of recruitment (December 2005), 415 packets were distributed to the agencies. An additional 172 packets were provided to 12 agencies who had distributed all of the original packets to allow for more subjects to participate prior to the December 2007 ending date. Based on the report of the focal contact persons, 414 survey packets were distributed by the agency focal persons to deploying aid workers.

**Table 4:** Association between risk/mitigating factors and burnout depersonalization (DP) and burnout emotional exhaustion (EE) and how this changes across time.

		AOR Post (95% CI)	AOR Follow-up (95% CI)	Type III p value
**Burnout DP**	**Family risk**	0.74 (0.36, 1.51)	0.37 (0.14, 1.01)	**0·022**
**Burnout EE**	**Health index**	0.82 (0.56–1.20)	0.57 (0.39-0.83)	0·085

AOR  =  adjusted odds ratio; CI  =  confidence interval.

p values are based on the Type III Wald chi-squared statistic.

p values <0·05 were considered statistically significant and are in bold type.

We selected and trained focal persons from each participating organization who were likely to have the most interaction with potential candidates for deployment (e.g., human resource staff). Training included a 1-day workshop in human subjects ethics, study methods, and how these focal persons would provide participants meeting the inclusion criteria with the invitation letters and pre-deployment questionnaires. The enrolment process included a standard oral introduction to the study by the focal person. Contact information was collected so that the study research coordinator could stay in communication with the participant and send the remaining assessments directly to the participants at Times 2 and 3.The enrollment period and follow-up covered December 2005–December 2009.

**Table 5:** Longitudinal multivariate generalized estimating equations model: demographic variables, exposure, organizational, and other risk and mitigating factors across time affecting burnout personal accomplishment (PA), depersonalization (DP), and emotional exhaustion (EE) subscales.

	Burnout (PA)		Burnout (DP)		Burnout (EE)	
Parameter	AOR (95%CI)	Type III p value	AOR (95%CI)	Type III p value	AOR(95%CI)	Type III p value
**Sex**Male versus female	0·97 (0·55–1·69)	0·907	0·55 (0·26–1·20)	0·123	0·45 (0·20–1·04)	0·062
**Age**	1·02 (0·98–1·06)	0·255	1·00 (0·96–1·05)	0·895	1·02 (0·97–1·08)	0·439
**Marital status**Not married versus married	1·04 (0·93–2·56)	0·881	0·77 (0·39–1·52)	0·459	0·98 (0·51–1·88)	0·951
**Job function**Non-manager versus head of mission	1·54 (0·93–2·56)	0·095	0·96 (0·48–1·94)	0·920	0·84 (0·41–1·73)	0·647
**Hardship assignment**						
Yes versus no	0·83 (0·40–1·71)		2·24 (0·94–5·33)		0·87 (0·34–2·19)	
Agency does not designate any hardship assignments versus no	0·69 (0·28–1·71)	0·384	2·21 (0·59–8·29)	0·101	1·93 (0·58–6·49)	0·077
Don't know versus no	1·26 (0·63–2·53)		3·46 (1·33–8·99)		2·35 (0·92–5·96)	
**History mental illness**No versus yes	0·87 (0·50–1·52)	0·626	0·76 (0·35–1·63)	0·486	1·14 (0·52–2·48)	0·749
**Social support**	0·95 (0·91–0·98)	**0·006**	0·96 (0·91–1·00)	0·075	0·98 (0·94–1·03)	0·516
**Trauma exposure category** *						
Traumatic stress category 2 versus 1	1·62 (0·96–2·74)	0·203	–	–	1·31 (0·69–2·46)	0·184
Traumatic stress category 3 versus 1	1·40 (0·61–3·18)				4·12 (1.27–13.33)	
**Positive NGO working experiences**Yes versus no	1·07 (1·01–1·14)	**0·025**	–	–	–	–
**Motivation**	0·93 (0·87–0·98)	**0·007**	–	–	–	–
**Chronic stress**Sum	1·02 (0·96–1·08)	0·575	–	–	1·11 (1·04–1·19)	**0·002**
**Health habits index**	–	–	0·86 (0·64–1·17)	0·353	–	–
**Extraordinary stress**	–	–	1·47 (1·17–1·83)	**0·005**	0·94 (0·75–1·07)	0·557
**Childhood trauma**	–	–	0·92 (0·58–1·45)	0·718	1·16 (0·76–1·77)	0·510
**Team cohesion field leader**	–	–	–	–	0·96 (0·90–1·02)	0·113

AOR  =  adjusted odds ratio; CI  =  confidence interval; NGO  =  non-governmental organization.

Each variable in the table was adjusted for all other variables in the table.

Time was also included as an adjustment variable in the analysis.

p values are based on the Type III Wald chi-squared statistic.

p values <0·05 were considered statistically significant and are in bold type.

* Trauma exposures are defined as follows:

Category 1 = 0 trauma events.

Category 2 = 1–4 traumatic events.

Category 3 = ≥5 traumatic events.

### Study instruments

In a separate article, we describe the characteristics of participants at pre-deployment and the methods in greater detail (accepted for publication, Eriksson C, Lopes Cardozo B, Foy D, et al., Traumatology, 2012) [9].

**Table 6:** Overview of longitudinal GEE models: all outcomes and selected variables with statistically significant associations across time.

Predictor	Anxiety	Depression	Psychological Distress	Burnout EE	Burnout PA	Burnout DP	Life satisfaction
Head of mission	–	–	–	–	–	–	Non-manager, less likely*
History of mental Illness	No history, less likely*	–	–	–	–	–	–
Marital status	–	–	Not married, less likely*	–	–	–	Not married, less likely*
NGO work experience	–	–	–	–	Better work experience, more likely*	–	–
NGO evaluation	More positive evaluation, more likely*	–	–	–	–	–	–
Exposure to extra ordinary stressors	–	More exposure, more likely*	–	–	–	More exposure, more likely*	–
Exposure to traumatic stressors	–	More exposure, more likely*	–	–	–	–	–
Chronic stress	–	More exposure, more likely†	–	More exposure, more likely†	–	–	–
Motivation	–	–	–	–	More motivation, less likely†	–	–
Social support	–	More support, less likely§	More support, less likely*	–	More support, less likely†	–	More support, more likely*
Alcohol use	–	–	–	–	–	–	More use, less likely†
Family risk	–	–	–	–	–	More family risk, more likely*	–
Adult Trauma	–	–	More exposure, more likely*	–	–	–	–
Coping Avoidance	_	_	–	_	_	_	Less avoidance more likely*

DP  =  depersonalization; EE  =  emotional exhaustion; NGO  =  non-governmental organization; PA  =  personal

accomplishment. AOR  =  Adjusted Odds Ratio; CI  = 95% Confidence Interval.

* p<0·05.

† p<0·01.

§ p<0·001.

The questionnaire was organized by a) pre-deployment predictors: demographics, pre-deployment preparation, daily living conditions (quality of housing, food sources, hygienic services, and political and social atmosphere within the host country), leisure-time options, motivational factors, communication with family and friends, organizational climate [10], NGO work experience and evaluation, psychiatric history (including early trauma [11]–[13], prior medication, and therapeutic interventions), number of missions, length of missions, and hardship assignments. b) Moderators during deployment: Chronic stressors, traumatic experiences (current and previous missions), social support[14], coping strategies[15], health habits [16], team cohesion during deployment [17], and availability of psychological support services (either offered by the organization or accessed informally in host country) during and post-assignment. c) Study outcomes included mental health measures: (anxiety, depression, and psychological distress [18], [19], [20]; burnout [21], [22], and burnout subscales of emotional exhaustion [EE] [21], [22], depersonalization [DP], personal accomplishment [PA], alcohol/drug use, life satisfaction[23], and job satisfaction [24]. Table S1 in the online content shows an overview of all the instruments. All responses were self-reported.

To determine the level of stress exposure pre-deployment and to differentiate the impact of stress caused by field experience on different outcome variables, we asked questions regarding past traumatic experiences. Participants were asked to respond to questions regarding their personal history (e.g., childhood physical or sexual abuse) and family-of-origin risk factors (e.g., exposure to parental and intimate partner domestic violence) [11]–[13]. Other questions regarding extraordinary stressors pre-deployment included questions about having experienced a life-threatening illness, having been in a serious car crash, or having been attacked or mugged [11].

NGO work experience questions explored NGO policies and their implementation (e.g., vacation or sick leave and satisfaction level with NGO-offered services). The NGO evaluation was a separate set of questions regarding a clear sense of mission, decision-making processes, organizational communication, and activity evaluation. This instrument was modified from a similar instrument the co-authors had developed during an earlier study among expatriate humanitarian aid workers [9].

### Stressors and traumatic experiences during deployment

Chronic stressors comprised questions regarding living conditions (housing and privacy, water and electricity availability/reliability), security concerns (threatening checkpoints, hostility from host country or beneficiaries), heavy workload and NGO's lack of recognition for accomplishments, and lack of communication. This instrument was modified from a similar instrument the authors had developed for previous studies [10], [2]. Trauma experiences included exposure to serious threatening events (e.g., being forced into unwanted sexual contact, threats of physical harm, having been kidnapped, murder of a colleague or family member, or deliberate destruction of home or office). This measure was adapted to better fit the specific context of international humanitarian aid workers, from similar instruments that were developed by the co-authors [10], [2].

The health habits index score [16] is constructed from an algorithm of health habits that included questions regarding eating, smoking, alcohol, drug and caffeine use, sleeping, and exercise habits.

In the Team Cohesion instrument, participants were asked to respond to questions regarding their experiences with headquarters leadership, field leadership, and with team members during their assignments only at Time 2, because post-deployment was the best point for participants to reflect on their experiences with their field team. The measure was adapted from the Team Cohesion Scale [17].

Participants were asked to report on how they cope with problems and troubles in their lives. The instrument was adapted from the *Coping Strategy Indicator*, which includes three subscales of coping strategies: Problem-Solving, Avoiding, or Social-Support Seeking [15]. The three items with the highest factor loadings on these subscales were used in this assessment. Average item scores for each of the three original CSI subscales were utilized in analyses.

## Outcomes

### Anxiety and depression

The Hopkins Symptom Checklist-25 (HSCL25) measured elevated symptoms associated with anxiety and depression and comprises 10 statements measuring elevated anxiety symptoms and 15 statements measuring elevated depression symptoms [18], [19], [20]. A 1·75 normed and validated cut-off score indicates a case of elevated anxiety or depression. However, these prevalences for anxiety, and depression are not equivalent to clinical diagnoses. The combined sub-scales of anxiety and depression of the HSCL-25 have also been used by some as a measure of psychological distress [25].

*Burnout* is a syndrome defined by three principal components of EE, DP, and diminished feelings of PA [21], [22]. Unlike major depressive disorder, which pervades all aspects of a person's life, burnout is a distinct work-related syndrome [21], [22]. Burnout is most likely to occur in jobs that require extensive care of others [21], [22]. The most commonly used tool for assessing burnout is the 22-item Maslach Burnout Inventory–Human Services Survey (MBI-HSS) [22]. Burnout is established by combining high scores in EE and DP and low score for PA [22]. We also examined how many participants met the cut-off scores for all three constructs to tabulate an overall case prevalence for burnout.

Five statements were asked concerning how workers feel about their life and are intended to provide a measure of life satisfaction [23]. Questions are on a seven-point Likert-type scale from “strongly disagree” to “strongly agree”. We created a mean score of the sum of five life-satisfaction questions.

Four questions asked how deployed staff felt about their jobs while on deployment to obtain descriptive information on job satisfaction; questions were on a five-point Likert-type format from “strongly disagree” to “strongly agree” [24]. We created a mean score of the sum of the four job satisfaction questions.

### Statistical analyses

Data were entered into Epi Info™ 2002 (CDC, Atlanta, Georgia). Data analyses were performed by using SPSS® 17·0 (IBM Corporation, Somers, New York) and SAS® 9·1 (SAS Institute Inc., Cary, North Carolina). Univariate and bivariate analyses using chi-square tests assessed differences and trends in categorical variables; comparable analyses based on Student's *t*-tests were used to assess continuous variables; and multivariate regression analysis was used to adjust for multiple risk factors and potential confounders. Analyses including more than one time point (i.e., pre-deployment and post-deployment or all three time points), accounted for the paired (for two time points), or longitudinal (for three time points) nature of the study. We performed bivariate regression analyses on all the variables and the outcomes of depression, anxiety, and burnout subscales to determine the main contributors to negative mental health outcomes and burnout at post-deployment and follow-up (Table S2).

We fitted generalized estimating equations (GEE) [26] longitudinal models for the outcomes of anxiety, depression, burnout EE, burnout DP, burnout PA, and life satisfaction. Adjustment for missing values was based on the method described in Diggle et al [27].

Screening tests were conducted to determine the most parsimonious multivariate model for each mental health outcome variable. These models were also used to investigate whether associations between outcome and predictive factors changed with time. If, after an initial assessment, the outcome variable varied significantly with time, we controlled for time and past time point assessments of psychological systems. If, however, the outcome variable did not vary significantly with time, then we did not control for time or past time point assessments, so we did not have to fit an unnecessarily complicated model. Thus, the final multivariate model adopted could be different for the different outcomes, although the initial variables considered in the modeling process were the same for all outcomes. The basic model used for each screening test was outcome  =  demographic variables + predictor + predictor × time. These screening tests allowed us to investigate the effects of the predictor variable, confounding factors, and interaction between predictor and time that measures the change in the association between outcome and predictor across time. If an interaction term between predictor and time was statistically significant, this interaction term as well as the predictor variable was included in the final model. The predictor variables and potentially confounding factors with p values <0·1 were selected for analysis in a final model. In addition, based on psycho-social theory, the following variables were included for consideration in the final longitudinal regression model: age, sex, marital status, job function (head of mission versus other), hardship deployment, and number of personally experienced trauma events during deployment (categorized into 0 events, 1–4 events, and ≥5 events) [3], [28].

## Results

The final number of participating organizations was 19. A total of 214 aid workers consented to participate in the Time 1 phase. One participant left the entire questionnaire blank and did not complete the non-response questions. One non-respondent filled out the non-response questions. Therefore, a total of 212 respondents were used for analysis. Of those, 170 (80%) aid workers completed the Time 2 questionnaire, and 154 (73%) also completed the Time 3 questionnaire. Out of the 19 humanitarian NGOs that originally agreed to participate, we did not receive any questionnaires from participants from two NGOs. A total of 10 questionnaires were sent out for distribution by the focal persons of these two organizations.

### Demographic characteristics

Of the respondents, 59% were female. The mean age of the respondents was 34 years. Forty percent were married or in a committed relationship and 54% were single. The educational level of respondents was high: 88% had a university or post-graduate degree. Demographic characteristics of the participants are shown in table 1.

### Mental health outcomes and life satisfaction at Times 1–3

**Figure 1:**
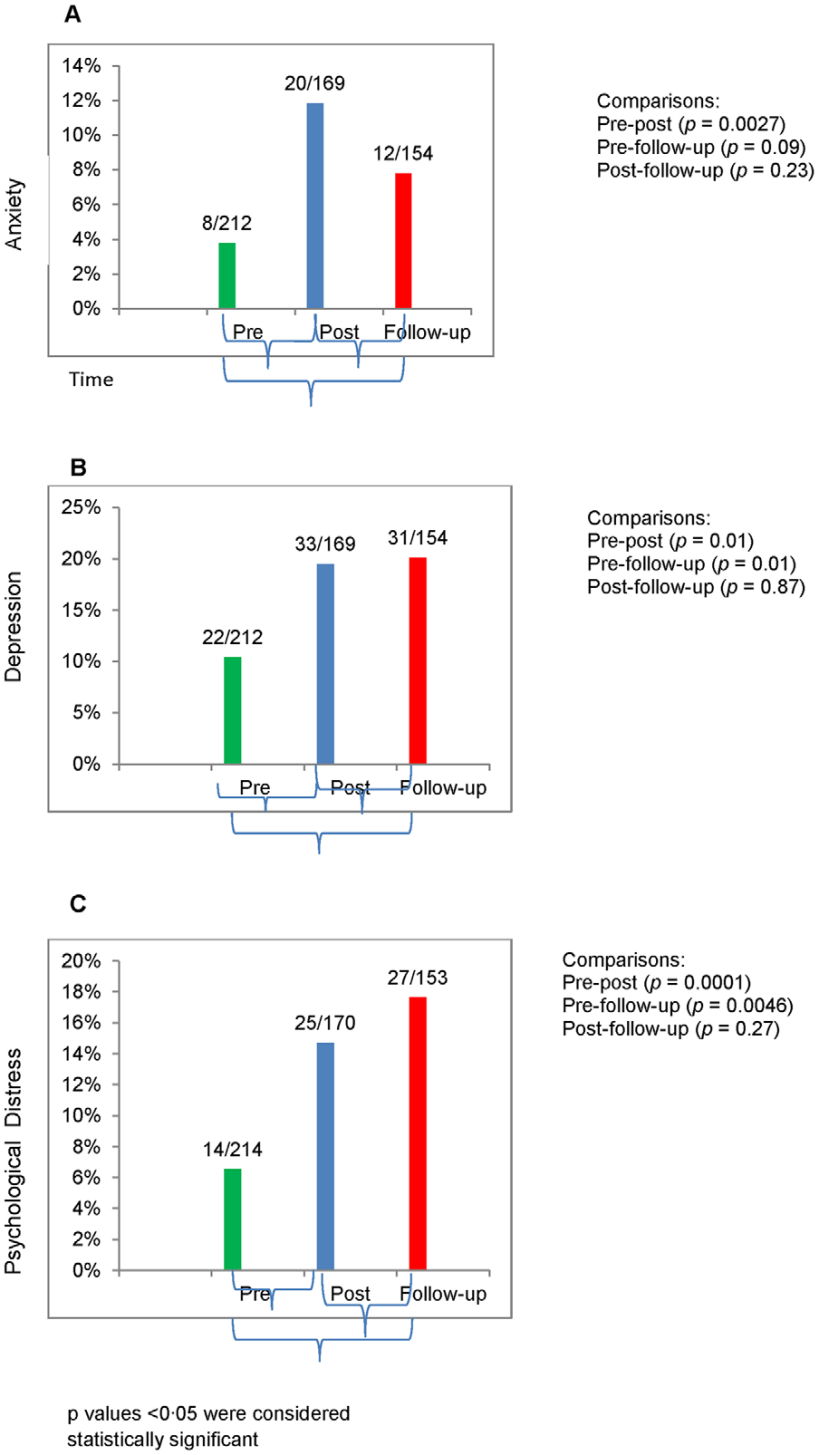
Mental Health Outcomes. Mental health outcomes at pre-deployment (N = 212), post-deployment (N = 169), and follow-up (N = 154) 3–6 months after returning from assignment.

Mental health outcomes of anxiety and depression based on cut-off measures demonstrated higher prevalences after field deployment than pre-deployment for anxiety [M(SD) pre = 1.28 (.25), post = 1.38 (.37), (p = 0·003], and for depression [M(SD) pre = 1.33 (.29), post = 1.51 (.36), p = 0·002], and, and for psychological distress [M(SD) pre = 1.32 (.25), post = 1.46 (.32), p = 0.0001]. See figure 1. This increase in prevalence tended to persist 3–6 months after post-deployment, and the prevalence of depression [M(SD) follow-up = 1.48 (.45), p = 0.008] and psychological distress [M(SD) follow-up = 1.42 (.39), p = 0.005] was significantly higher at follow-up than at pre-deployment.

**Figure 2:**
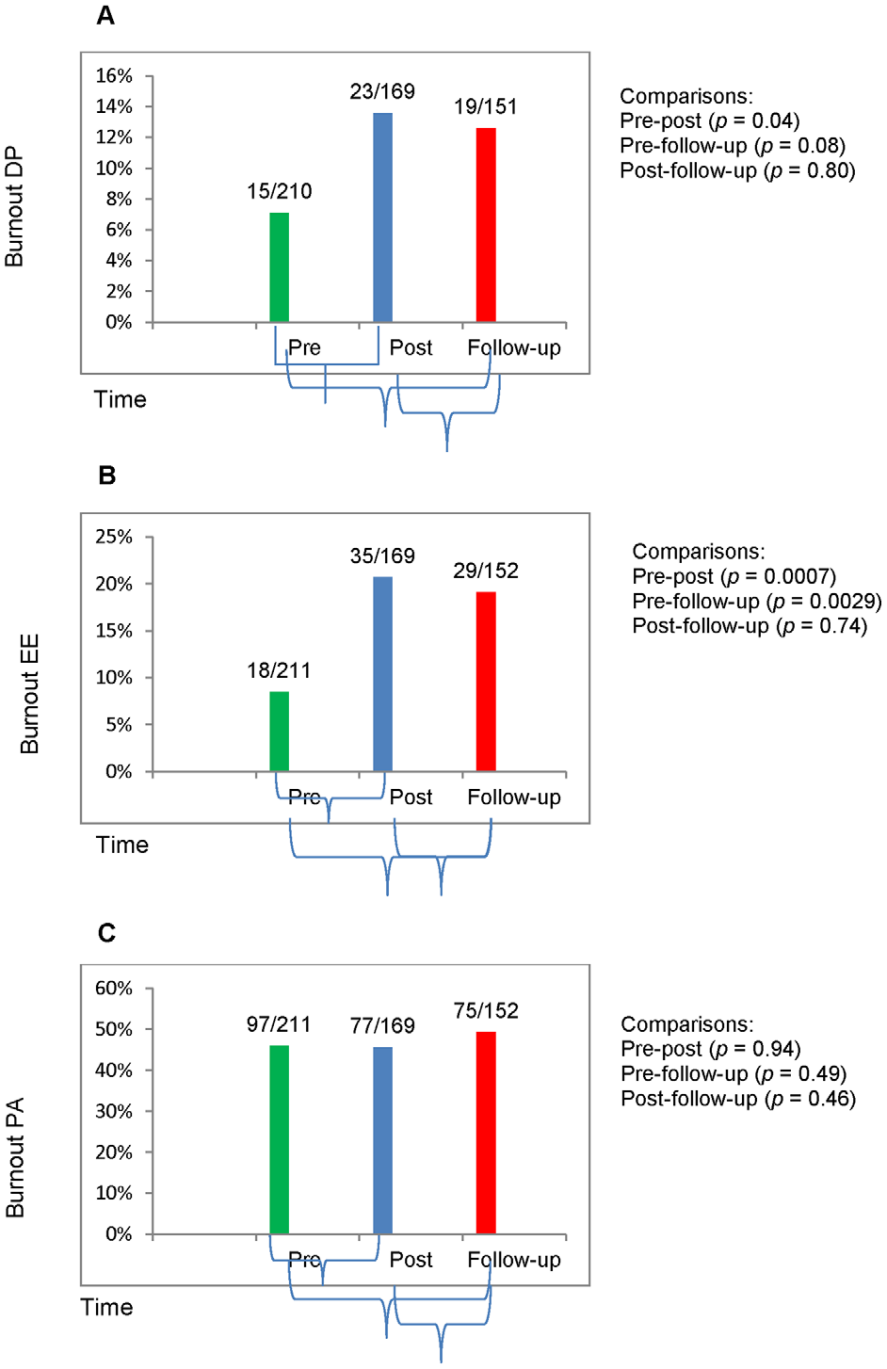
Burnout Outcomes. Burnout outcomes at pre-deployment (N = 210 or 211), post-deployment (N = 169), and follow-up (N = 151 or 152) 3–6 months after returning from assignment.

Trends for burnout cases on depersonalization (DP) (p = 0·037) and emotional exhaustion (EE) (p = 0·001) demonstrated higher levels post-deployment (figure 2). We also identified an increase in EE prevalence that persisted 3–6 months after post-deployment (p = 0·003). Five participants met all three criteria for burnout before deployment and 6 months after deployment, but the difference was not statistically significant.

The mean score of the sum of the life satisfaction items was significantly lower at follow-up than at pre-deployment (p = 0·012). No significant difference existed between life satisfaction at pre-deployment compared with post-deployment (Figure S1).

#### Bivariate Analyses

Intercorrelations between outcome variables of anxiety, depression, EE, DP, and personal achievement (PA) burnout subscales were calculated for all three time points (Table S2). Anxiety and depression were highly correlated at each of the assessments (*r*  = .532 to.656). In addition, correlations between mental health outcomes and burnout subscales of EE and DP ranged from *r*  = .154 to.440. PA was not significantly correlated with anxiety or depression.

The depression and anxiety outcome scores, EE, DP, and PA were cross-tabulated with demographic variables, organizational-related factors, trauma exposure, chronic stress, and health behavioral and personal factors (e.g., motivation, spirituality, and coping) (Table S3).

At **pre-deployment**, lower levels of education was associated with higher levels of burnout of the PA subscale (p = 0·025). Not being a manager was associated with a lower level of burnout on the PA sub-scale (p = 0·037). Not having a history of mental illness was significantly correlated with less depression (p = 0·002) and anxiety (p = 0·045). Social support was significantly correlated with less depression (p = 0·015) and lower levels of burnout on the PA subscale (p = 0·002). Lower motivation was correlated with higher levels of burnout on the PA subscale (p = 0·030). Low avoidance on the coping scale was associated with a higher risk for depression (p = 0.010). A higher health habits index score was correlated with a lower risk for depression (p = 0·033). A history of childhood trauma was associated with higher levels of depression (p = 0·050) and anxiety (p = 0·057). Having experienced extraordinary stressors pre-deployment was associated with higher levels of depression (p = 0·0003), anxiety (p = 0·028), and burnout DP (p = 0·053). (Table S3).

At **post deployment**, not having a history of mental illness was significantly correlated with a lower risk for depression (p = 0.026) and anxiety (p = 0.013). Social support was significantly correlated with a lower risk for depression (p = 0·001). Lower motivation was correlated with a higher risk for depression (p = 0·038), burnout EE subscale (p = 0·028), and with the burnout PA subscale (p = 0·045).

A better NGO work experience (p = 0·028) was associated with higher levels of burnout on the PA subscale. Field leader team cohesion (p = 0·010), and using social support as coping mechanisms (p = 0·030) were both associated with a lower risk for burnout on the PA subscale. A higher health habits index score was correlated with a lower risk for anxiety (p = 0·006). Chronic stressors during deployment were correlated with higher levels of depression (p = 0·010), anxiety (p = 0·038), and the burnout EE (p = 0·001) and DP (p = 0·003) subscales. Higher levels of experienced trauma events during deployment were correlated with a higher risk for burnout on the DP subscale (p = 0·050) (Table S3).

**At follow-up**, not having a history of mental illness was significantly correlated with lower risk for anxiety (p = 0.005). Social support was significantly correlated with lower risk for depression (p = 0·037) and burnout on the PA subscale (p = 0·009). The health habits index score was correlated with a lower risk for anxiety (p = 0.006) and burnout on the EE subscale (p = 0·035). (Table S3).

### Generalized Estimating Equations (GEE)

Results of the GEE models are presented in tables for the mental health outcome variables of depression and anxiety and for the three burnout subscales.

The effect of deployment-related chronic stress (p = 0·005), and extraordinary stress exposure (p = 0·041) at pre-deployment on depression changed significantly across the three time points (table 2). The risk for depression increased with higher scores of exposure to deployment chronic stress (e.g. excessive workload, conflict with colleagues, lack of recognition, etc.) at both post-deployment (AOR = 1·11;[CI] 1.02–1.20) and follow-up (AOR = 1·03;[CI] 0.93–1.14). However, compared at the same chronic stress score (mean = 13·4), a higher risk for depression occurred after the humanitarian aid workers returned from assignment. As indicated by Table 2, it shows that there was a significant difference (p = 0.041) in the effect of the predictor–extraordinary stress–on depression over time (i.e. post-deployment vs. pre-deployment; and follow-up vs. pre-deployment.

The association between depression and traumatic exposure during deployment also changed with time (p = 0.055). The odds ratio is not constant for the deployment traumatic exposure score across time. Table 2 demonstrates that the odds ratios are higher at post-deployment than at follow-up; thus the effect of trauma exposure on depression is significantly greater post-deployment but tends to diminish three to six months after deployment.

Table 2 indicates that the health habits index variable associated with anxiety varied with time; however, this variation was not statistically significant. Variables associated with the psychological distress measure did not change across time.

In the GEE model for depression, we determined that social support was significantly associated with experiencing depression (AOR = 0·89; [CI] 0.84–0.95) (table 3).

Not having a history of mental illness was significantly associated with lower risk for anxiety (AOR = 0·24; [CI] 0.08–0.69). Having a higher mean score for a positive evaluation of the NGO was associated with higher risk for anxiety (AOR = 1.09; [CI] 1.01–1.19) (table 3). None of the other variables displayed in table 3 are significantly associated with depression or anxiety.

Not being married and social support were significantly associated with a lower risk for psychological distress (AOR = 0.31; [CI] 0.15–0.65) and (AOR = 0.91; [CI] 0.85–0.97). Having experienced trauma as an adult was significantly associated with higher risk for psychological distress (AOR = 2.71 [CI] 1.35–5.48) (table 3).

Table 4 reveals that the relationship between family risk factors and burnout DP varied significantly over time (p = 0·022). Higher family risk was associated with higher risk for the burnout DP subscale at pre-deployment, but this risk was less important at post-deployment and follow-up, compared with the risk at pre-deployment (p = 0·022).

Having experienced higher levels of pre-deployment extraordinary stress was significantly (AOR = 0·47; [CI] 1.17–1.83) associated with higher burnout on the DP subscale (table 5). Having had a positive experience with the NGO with which the participant was working was significantly associated with lower levels of burnout on the PA subscale (AOR = 1.07; [CI] 1.08–1.14). Having less social support (AOR = 0·95; [CI] 0.91–0.98) and lower motivation levels (AOR = 0·93; [CI] 0.87–0.98) were significantly associated with higher levels of burnout on the PA subscale (table 5). Having experienced more chronic stress (AOR = 1.11; [CI] 1.04–1.19) was significantly associated with higher levels of burnout on the EE subscale (table 5).

Tables 2 and 4 provide results for variables in which the association between outcome and predictor changed with time. In these cases, a separate odds ratio (OR) exists for each time point. Tables 3 and 5 display results for variables in which the association between outcome and predictor did not change across time (hence, one estimated odds ratio exists for all three time points).

When adjusted for other variables, Life satisfaction was significantly lower at post-deployment and follow-up, compared with pre-deployment levels (p = 0.002) (Table S4). Not being married was significantly associated with lower levels of life satisfaction than being married (p = 0.014). Job function was significantly associated with life satisfaction (p = 0·014), with respondents who were not in a management function reporting lower levels of life satisfaction than respondents in managerial functions. Participants who reported having more social support also had significantly higher levels of life satisfaction (p = 0·021). Respondents who reported less alcohol use had higher levels of life satisfaction than those who reported more alcohol use (p = 0.003) (Table S3). Respondents who used a lot of avoidance as coping mechanism had lower levels of life satisfaction (p = 0.015) (Table S4).

Job satisfaction was only measured at post-deployment. Regression analysis at post-deployment revealed that participants who had a more positive evaluation of their NGO reported significantly higher job satisfaction at post-deployment (p = 0·001). (Table S5).

We are also providing an overview of all longitudinal GEE models of all outcomes and those variables that were statistically significant across time (Table 6).

## Discussion

Our study indicates that humanitarian aid workers are at increased risk for depression and burnout EE after they returned from deployment, and this risk did not diminish 3–6 months after assignment completion. They also had an increase in anxiety and burnout DP immediately post-deployment, but this risk did not persist 3–6 months after assignment completion. Also of concern is that aid workers had lower levels of life satisfaction at follow-up months after their deployment, compared with pre-deployment.

We identified factors that might have contributed to an increased risk for mental illness and burnout and lower life satisfaction. We also identified factors that seem to be a protective effect against the risk for experiencing mental illness or burnout across time or resulted in higher levels of life and job satisfaction.

Persons with a history of mental illness might be in need of special counseling and support when NGOs consider deployment. These candidates might be at increased risk for suffering from anxiety or depression and burnout DP as a consequence of deployment. Those who have experienced crucial personal stressors before deployment (e.g., having been in a serious car crash or having had a serious physical illness) may also be at increased risk for burnout DP. In addition people who had a history of domestic violence or similar experiences before deployment are at higher risk for psychological distress.

Participants with strong social support networks were less likely to suffer negative mental health consequences from their deployment. Workers with strong social support networks were less likely to suffer from depression, psychological distress, or burnout related to PA, and they had higher levels of life satisfaction throughout their deployment. These findings lends scientific support for the recommendations that peer support networks are beneficial for aid workers during or after their deployment [3]. Workers who were married also had higher levels of life satisfaction. However, those respondents who were not married were at lower risk for psychological distress. Although being married may provide more support and satisfaction it also comes with certain responsibilities which could cause worries and stress during deployment.

We cannot confirm that aid workers who scored higher on health habits (e.g., eating healthier, smoking less, and sleeping and exercising more) were less likely to be at risk for mental illness or burnout symptoms. Other studies of health professionals have reported that health habits are related to job burnout and might help prevent it [29], [30]. Unlike the Radostina and Muros study [31], we did not find any gender differences among aid workers in burnout outcomes. However, our respondents who reported drinking more alcohol had lower levels of life satisfaction.

Aid workers who had high levels of motivation were less likely to suffer from burnout as measured on the PA subscale. Because scientific studies of humanitarian aid workers are lacking, this is the first time that this specific association has been reported. That persons with high levels of motivation to do this kind of work in difficult circumstances are less at risk for burnout makes sense. The burnout concept was developed around the idea that it can lead to a lack of job motivation, but the reverse might also be true [21].

A reportedly better experience in working with an NGO was associated with higher levels of burnout on the PA scale. A more positive evaluation of working with an NGO was also associated with higher levels of anxiety cases. These findings seem somewhat counter-intuitive. However, the positive experience of working with the NGO might put the responsibility more on the worker if tasks do not go as well as planned. Similarly, a more positive NGO evaluation might mean that respondents took responsibility for failure on themselves, or maybe they believe they are not living up to the organization's goals.

Respondents who were working in managerial positions were more likely to have higher levels of life satisfaction. Other studies have also found that employees have higher levels of job satisfaction when they have more autonomy and control over their work [32].

Chronic stressors during deployment are inherent to working in humanitarian emergencies. We determined that more exposure to chronic stress was related to higher risk for depression and burnout at the EE scale. However, chronic stressors can be lessened by improving accommodation facilities whenever possible, facilitating as much access to communication with home as possible [6], regulating workload of staff, improving management directions to the teams, and providing recognition by the organization for optimal work performance.

Participants who had experienced more traumatic stress during deployment were more likely to have higher levels of depression. The association of experiencing traumatic events and Posttraumatic Stress Disorder (PTSD) is well-known. However, the relation between traumatic stress experiences and depression has not been explored extensively. In our study, humanitarian aid workers who were exposed to a higher number of traumatic events were at an increased risk for depression, but this risk was more prominent at post-deployment than at follow-up, meaning that the effect of the same level of traumatic stress exposure became less important with time.

Respondents who had been exposed during their childhood to family risk factors (e.g., physically abusive parents or in other ways exposed to violent behavior, parents' or siblings' death, or divorced parents) were at risk for suffering burnout DP. However, this risk was more influential on outcomes at pre-deployment compared with post-deployment and follow-up. One explanation might be that these difficult childhood experiences might have prepared these aid workers to better handle deployment.

As expected, participants who had a more positive evaluation with their NGO had higher levels of job satisfaction at post-deployment. Working in a hardship assignment was unassociated with an increase in anxiety, depression, or burnout. This finding indicates that, despite the hardship of working in a dangerous and uncomfortable environment, such work did not contribute to more stress-related mental illness or burnout.

Our study had certain limitations. One limitation is related to the sampling of agencies. Despite intense efforts, the majority of agencies contacted from the initial list of possible organizations declined participation or did not respond to the inquiry. This may indicate that the agencies choosing to participate in this study represent a sample of agencies with adequate resources and/or a particular interest the research topic, and this may influence how they select and screen their staff. However, agencies that did not agree to participate may have less concern and support for their staff, potentially resulting in underestimating associations between stress and mental health in humanitarian aid workers.

Selection bias might exist because we cannot know with certainty if the organization's focal persons handed out the questionnaires to all workers who met the inclusion criteria pre-deployment. However, all focal persons received training before study commencement, and the enrolment process included a standard oral introduction to the study by the focal person. They had regular contact with the research coordinator and were able to ask questions whenever needed. Furthermore, each agency had different logistics regarding how staff were recruited and deployed. This made measuring the initial response rates from the aid workers difficult. However, of those aid workers who returned the pre-deployment questionnaire, 80% also returned the post-deployment and 73% the follow-up questionnaire. Respondents to this study were asked to return the follow-up questionnaire 3 to 6 months after deployment, which was a relatively long period during which mental health could have changed. The reason we gave them a range of time to return the questionnaire was to provide the aid workers with some flexibility in time in the hope this would increase compliance. The follow-up time of the study was limited to 6 months after deployment. Therefore we cannot provide any results on long term consequences of deployment of international humanitarian aid workers.

Our study did not include a measure of resiliency because the concept of resiliency and adequate instruments to measure this were not well defined at the outset of this study. Future studies among aid workers should also emphasize the implications of resilience. Our findings have important ramifications for what humanitarian organizations can do to diminish the risk for experiencing mental illness or burnout during deployment, including the following:

- Screen candidates for a history of mental illness and family risk factors pre-deployment and provide expatriate employees psychological support during deployment and after the assignment is completed. Although possibly controversial given the considerable stigma associated with mental illness, screening allows organizations to alert candidates to the risks associated with deployment and to consider means for managing and supporting such workers during and after their employment.
- Staff should be informed that a history of mental illness and family risk factors may create increased risk for psychological distress during deployment.
- Provide the best possible living accommodations, workspace, and reliable transportation.
- Ensure, when possible, a reasonable workload, adequate management, and recognition for achievements.
- Encourage involvement in social support and peer networks.
- Institute liberal telephone and Internet use policies, paid by the organization will help increase social support networks of deployed staff.

## Supporting Information

Figure S1 **Life satisfaction at pre, post, and follow-up.**
(TIFF)

Table S1 **Overview of instruments.**
(DOC)

Table S2 **Intercorrelations for scores of outcome variables at 3 time points.**
(DOC)

Table S3 **Unadjusted bivariate analysis of mental health outcomes and burnout subscales versus risk/mitigating factors.**
(DOC)

Table S4 **Longitudinal multivariate generalized estimating equations model: demographic variables, exposure, organizational and other risk and mitigating factors affecting life satisfaction.**
(DOC)

Table S5 **Job satisfaction versus risk/mitigating factors at post-deployment.**
(DOC)
