# Supplementary figures and images for: Psychological Distress, Depression, Anxiety, and Burnout among International Humanitarian Aid Workers: A Longitudinal Study
Source: PLoS One. 2012 Sep 12;7(9):e44948. doi: 10.1371/journal.pone.0044948 (PMC3440316; doi:10.1371/journal.pone.0044948)

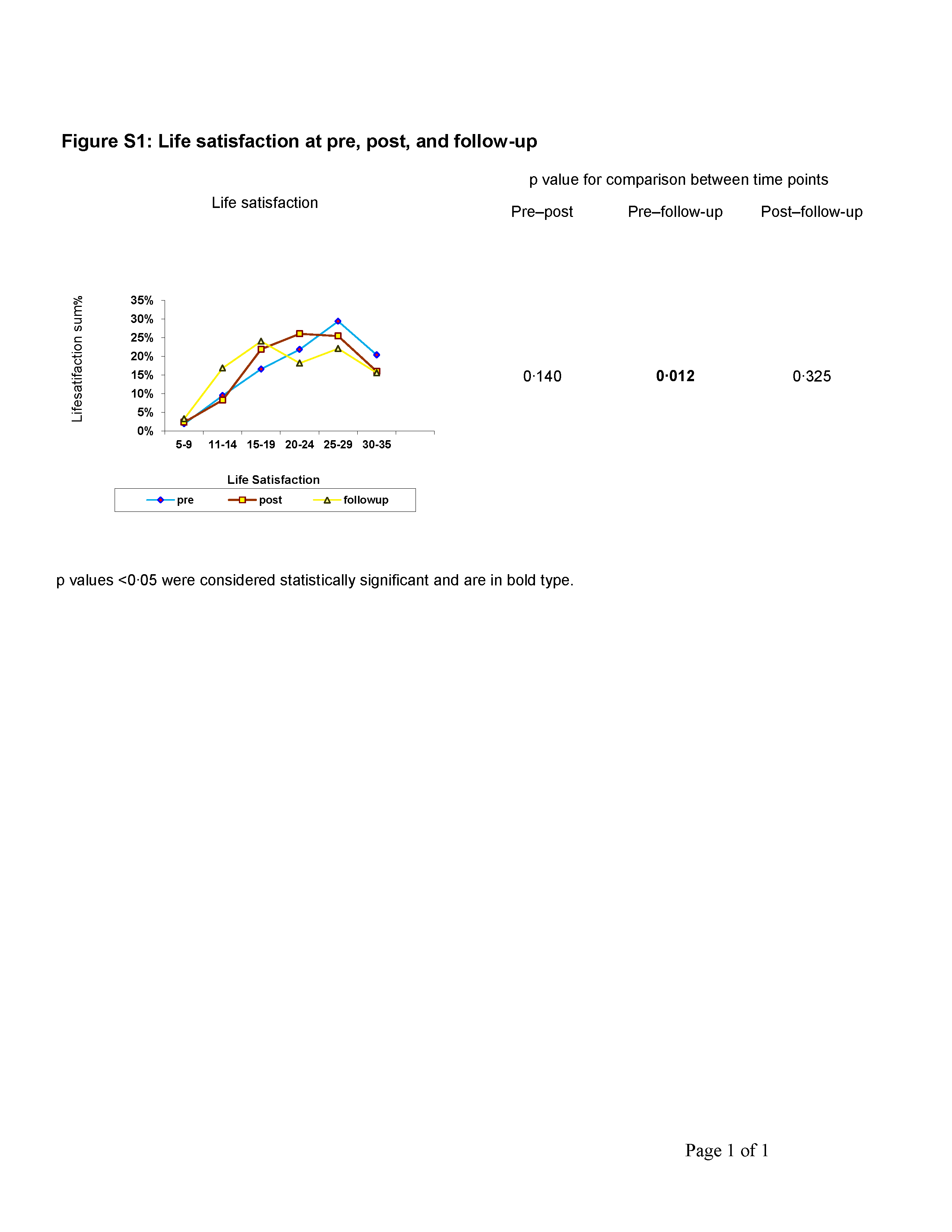

Supplement: Figure S1 — Life satisfaction at pre, post, and follow-up. (TIFF) [file pone.0044948.s001.tif]
