# Supplementary material for: Psychological Distress, Depression, Anxiety, and Burnout among International Humanitarian Aid Workers: A Longitudinal Study
Source: PLoS One. 2012 Sep 12;7(9):e44948. doi: 10.1371/journal.pone.0044948 (PMC3440316; doi:10.1371/journal.pone.0044948)
Supplement: Table S1 — Overview of instruments. (DOC) [file pone.0044948.s002.doc]

**Table S1: Overview of instruments**

|  | | Instrument | Reference for Instrument | # items | Pre | Post | FU |
| --- | --- | --- | --- | --- | --- | --- | --- |
| **Predictors** | |  |  |  |  |  |  |
| 1. Demographics | | Adapted from CDC-Kosovo study | Lopes Cardozo B, Holtz T, Kaiser R, et al. (2005) Mental health of expatriate and Kosovar Albanian humanitarian aid workers. Disasters 29: 152–70. | 15 | Y  15 | Y  11 | Y  8 |
| 1. Deployment experience | | Adapted from CDC-Kosovo study | Lopes Cardozo B, Holtz T, Kaiser R, et al. (2005) Mental health of expatriate and Kosovar Albanian humanitarian aid workers. Disasters 29: 152–70. | 5 | Y  5 | Y  4 | N |
| 1. Organizational support | | Adapted from Headington-WVI study | Eriksson, C.B., Bjorck, J., & Abernethy, A. (2003). Occupational Stress, trauma, and adjustment in expatriate humanitarian aid workers (pp. 68-100). In J. Fawcett (Ed.), Stress and Trauma Handbook: Strategies for Flourishing in Demanding Environments. Monrovia, CA: World Vision International. | 3 | Y  3 | Y  2 | Y  3 |
| 1. Treatment for Mental Health Problems | Adapted from CDC-Kosovo study | | Lopes Cardozo B, Holtz T, Kaiser R, et al. (2005) Mental health of expatriate and Kosovar Albanian humanitarian aid workers. Disasters 29: 152–70. | 2 | Y  2 | N | N |
| 1. History of using medications/alcohol | Adapted from CDC-Kosovo study | | Behavioral Risk Factor Surveillance System Questionnaire 2000. Atlanta, GA: National Center for Chronic Disease Prevention and Health Promotion, Centers for Disease Control and Prevention, U.S. Department of Health and Human services, may be accessed at http://www.cdc.gov/brfss/ | 5 | Y  5 | Y  5 | Y  5 |
| 1. Prior relational trauma/family risk factors | Adapted from Headington-WVI study:  Rutter’s Risk Factors | | Resnick, H. S. (1996). Psychometric review of Trauma Assessment for Adults (TAA). In B. H. Stamm (Ed.). Measurement of stress, trauma, and adaptation. Lutherville, MD: Sidran Press.  Straus, M. A. (1979). Measuring intrafamily conflict and violence: The Conflict Tactics Scale. Journal of Marriage and the Family, 41, 75-88.  Knutson, J. (1988). Physical and sexual abuse in children. In D. Routh (Ed.), Handbook of Pediatric Psychology. New York: Guilford.  Rutter, M., Silberg, J., O'Connor, T., & Simonoff, E. (1999). Genetics and child psychiatry: I. Advances in quantitative and molecular genetics. *Journal of Child Psychology and Psychiatry, 40*, 3–18. | 7 | Y | N | N |
| 1. Other earlier trauma |  | | Widom, Cathy Spatz; Weiler, Barbara Luntz; Cottler, Linda B  Childhood victimization and drug abuse: a comparison of prospective and retrospective findings.  Journal of Consulting and Clinical Psychology, 67(6): pp. 867-880, December 1999  Resnick, Heidi S; Kilpatrick, Dean G; Lipovsky, Julie A  Assessment of rape-related posttraumatic stress disorder: stressor and symptom dimensions.  Psychological Assessment, 3(4): pp. 561-572, 1991 | 10 | Y | N | N |
| 1. Motivational factors | Antares - Sept. 2003 Motivation items | | Created by CDC-Antares research working group | 10 | Y  10 | Y  10 | Y  10 |
| Total items predictors |  | |  | 57 | 57 | 32 | 26 |
| **Moderators** |  | |  |  |  |  |  |
| 1. Health habits | Adapted from Headington-WVI study | | Behavioral Risk Factor Surveillance System Questionnaire 2000. Atlanta, GA: National Center for Chronic Disease Prevention and Health Promotion, Centers for Disease Control and Prevention, U.S. Department of Health and Human services, may be accessed at http://www.cdc.gov/brfss/ | 7 | Y  7 | Y  7 | Y  7 |
| 1. Team cohesion (peer, leader, supervisor) | Team Cohesion Scale | | Bliese, P.D., & Halverson, L. (1996) Vertical and Horizontal Team Cohesion Scale. Journal of Applied Social Psychology, 26, 1171-1189. | 16 | N | Y  16 | N |
| 1. Coping with stress | Adapted from Amirkhan and “expert response” | | | Amirkhan JH.  J Pers Assess. 1994, April;62(2):242-61. |  | | --- | --- |   Criterion validity of a coping measure. | 15 | Y  15 | Y  15 | Y  15 |
| 1. Social support | Social Provisions Scale – Cutrona & Russell | | Cutrona, C. E. (1989). Ratings of social support by adolescents and adult informants: Degree of correspondence and prediction of depressive symptoms. Journal of Personality and Social Psychology, 57, 723-730.  Cutrona, C. E. & Russell, D. W. (1987). The provisions of social relationships and adaptation to stress. Advances in Personal Relationships, 1, 37-67. | 16 | Y  16 | Y  16 | Y  16 |
| 1. Chronic stress and   Traumatic Stress  Exposure | Adapted from Headington-WVI study and CDC-Kosovo Study | | Eriksson, C. B., Vande Kemp, H., Gorsuch, R., Hoke, S., & Foy, D. (2001). Trauma exposure and PTSD symptoms in international relief and development personnel. Journal of Traumatic Stress, 14, 205-212.    Lopes Cardozo B, Holtz T, Kaiser R, et al. (2005) Mental health of expatriate and Kosovar Albanian humanitarian aid workers. Disasters 29: 152–70. | 25  34 | N  N | Y  25  Y  34 | N  N |
| Total items moderators |  | |  | 117 | 42 | 117 | 42 |
| **Outcomes** |  | |  |  |  |  |  |
| 1. General health and health complaints. | General Health and Subjective Health Complaints | | Aasland, O.G., Olff, M., Falkum, E., Schweder, T., & Ursin, H. (1997). Health complaints and job stress in Norwegian physicians: The use of an overlapping questionnaire design. Social Science & Medicine, 45(11), 1615-1629.  Eriksen, H.R., Ihlebaek, C., & Ursin, H. (1999). A scoring system for subjective health complaints (SHC). Scandinavian Journal of Public Health, 1, 63-72. | 3  10 | Y  3  10 | Y  3  10 | Y  3  10 |
| 1. Substance abuse | LASC | | King, L., King, D., Leskin, G., & Foy, D. (1995). The Los Angeles symptom checklist: A self-report measure of posttraumatic stress disorder. Assessment, 2, 1-17. | 2 | Y  2 | Y  2 | Y  2 |
| 1. Depression 2. Anxiety | Hopkins SCL -25 | | Derogatis LR, Lipman RS, Rickels K, Uhlenhuth EH, Covi L**.** The Hopkins Symptom Checklist (HSCL): a self-report symptom inventory. *Behav Sci.*1974; 19:1-15.  Mollica RF, Wyshak, de Marneffe G, Khuon D, & Lavelle J. Indochinese versions of the Hopkins Symptom Checklist-25: a screening instrument for the psychiatric care of refugees. *Am J Psychiatry.* 1987; 144:497-500. | 15  10 | Y  15  Y  10 | Y  15  Y  10 | Y  15  Y  10 |
| 1. Burnout | Maslach Burnout Inventory - Human Services Survey | | Maslach, C. & Jackson, S. E. (1996). Maslach Burnout Inventory – Human Services Survey (MBI-HSS). In C. Maslach, S. E. Jackson, & M. P. Leiter, MBI Manual (3^rd^ Ed.). Palo Alto, CA: Consulting Psychologists Press. | 27 | Y  27 | Y  27 | Y  27 |
| 1. Life satisfaction | Satisfaction with Life Scale | | Diener, E., Emmons, R. A., Larsen, R. J., & Griffin, S. (1985). The Satisfaction with Life Scale. Journal of Personality Assessment, 49, 71-75. | 5 | Y  5 | Y  5 | Y  5 |
| 1. Job satisfaction | Items from Brayfield & Rothe | | Brayfield, A.H. & Rothe, H.F. (1951). An index of job satisfaction. Journal of Applied Psychology, 35, 307-311.  As cited in:  Price, J.L. & Mueller, C.W. (1986). Handbook of Organizational Measurement. Marshfield, MA: Pitman. | 4 | N  4 | Y  4 | N  4 |
| TOTAL items outcomes |  | |  | 93 | 89 | 93 | 89 |
| **GRAND TOTAL ITEMS** |  | |  | **267** | **188** | **242** | **157** |
