# Supplementary material for: Psychological Distress, Depression, Anxiety, and Burnout among International Humanitarian Aid Workers: A Longitudinal Study
Source: PLoS One. 2012 Sep 12;7(9):e44948. doi: 10.1371/journal.pone.0044948 (PMC3440316; doi:10.1371/journal.pone.0044948)
Supplement: Table S2 — Intercorrelations for scores of outcome variables at 3 time points. (DOC) [file pone.0044948.s003.doc]

**Table S2: Intercorrelations for scores of outcome variables at 3 time points**

**Measure 1 2 3 4 5**

**Pre-deployment**

| 1. HSC Anxiety | -- |  |  |  |  |
| --- | --- | --- | --- | --- | --- |
| 2. HSC Depression | .532** | -- |  |  |  |
| 3. MBI Emotional Exhaustion | .178** | .302** | -- |  |  |
| 4. MBI Depersonalization | .154* | .227** | .583** | -- |  |
| 5. MBI Personal Accomplishment | -.054 | -.091 | .145* | .080 | -- |

Post-deployment

| 1. HSC Anxiety | -- |  |  |  |  |
| --- | --- | --- | --- | --- | --- |
| 2. HSC Depression | .598** | -- |  |  |  |
| 3. MBI Emotional Exhaustion | .352** | .365** | -- |  |  |
| 4. MBI Depersonalization | .199** | .232** | .556** | -- |  |
| 5. MBI Personal Accomplishment | -.063 | -.087 | .116 | -.034 | -- |

Follow-up

| 1. HSC Anxiety | -- |  |  |  |  |
| --- | --- | --- | --- | --- | --- |
| 2. HSC Depression | .656** | -- |  |  |  |
| 3. MBI Emotional Exhaustion | .434** | .440** | -- |  |  |
| 4. MBI Depersonalization | .269** | .266** | .479** | -- |  |
| 5. MBI Personal Accomplishment | .019 | -.156 | .066 | -.030 | -- |

*Note:* HSC = Hopkins Symptom Checklist; MBI = Maslach Burnout Inventory.

*p < .05. ** p < .01.
