# Supplementary material for: Psychological Distress, Depression, Anxiety, and Burnout among International Humanitarian Aid Workers: A Longitudinal Study
Source: PLoS One. 2012 Sep 12;7(9):e44948. doi: 10.1371/journal.pone.0044948 (PMC3440316; doi:10.1371/journal.pone.0044948)
Supplement: Table S3 — Unadjusted bivariate analysis of mental health outcomes and burnout subscales versus risk/mitigating factors. (DOC) [file pone.0044948.s004.doc]

**Table S3: Unadjusted bivariate analysis of mental health outcomes and burnout subscales versus risk/mitigating factors***

| **Outcome** | **Risk/mitigating factors** | **Pre** | | | **Post** | | | **Follow-up** | | |
| --- | --- | --- | --- | --- | --- | --- | --- | --- | --- | --- |
|  |  | **OR** | **95% CI** | **p value** | **OR** | **95% CI** | **p value** | **OR** | **95% CI** | **p value** |
|  | **Demographics** |  |  |  |  |  |  |  |  |  |
|  | Age |  |  |  |  |  |  |  |  |  |
|  | Sex |  |  |  |  |  |  |  |  |  |
| Burnout PA | Education  (Ref=High School) |  |  |  |  |  |  |  |  |  |
|  | BS/MS | 0.87 | 0.37, 2.08 | **0·025** |  |  |  |  |  |  |
|  | Ph.D or Higher | 0.33 | 0.12, 0.93 |  |  |  |  |  |  |
|  | Marital status |  |  |  |  |  |  |  |  |  |
| Burnout EE | Job function  Head of Mission vs. Non Manager (ref.) | 2.57 | 0.97, 6.83 | **0·058** |  |  |  |  |  |  |
| Burnout PA | Job function  Head of Mission vs. Non Manager (ref.) | 0.52 | 0.28, 0.96 | **0·037** |  |  |  |  |  |  |
|  | Current employment |  |  |  |  |  |  |  |  |  |
|  | Hardship assignment |  |  |  |  |  |  |  |  |  |
| Depression | History of mental illness  No versus yes | 0.25 | 0.10, 0.62 | **0·002** | 0.39 | 0.17, 0.89 | **0.026** | 0.66 | 0.27, 1.60 | 0.356 |
| Anxiety | History of mental illness  No versus yes | 0.23 | 0.06, 0.97 | **0·045** | 0.29 | 0.11, 0.77 | **0.013** | 0.18 | 0.05, 0.60 | **0.005** |
|  | **Organization** |  |  |  |  |  |  |  |  |  |
| Depression | Social support | 0.90 | 0.83, 0.98 | **0·015** | 0.90 | 0.85, 0.96 | **0·001** | 0.94 | 0.88, 1.00 | **0·037** |
| Burnout PA | Social support | 0.92 | 0.87, 0.97 | **0·002** | 0.95 | 0.91,1.00 | 0·061 | 0.93 | 0.88, 0.98 | **0·009** |
|  | Organizational support |  |  |  |  |  |  |  |  |  |
| Burnout PA | NGO work experience |  |  |  | 1.09 | 1.01, 1.08 | **0·028** |  |  |  |
|  | Team cohesion  (headquarters lead) |  |  |  |  |  |  |  |  |  |
| Burnout PA | Team cohesion  (field lead) |  |  |  | 0.92 | 0.87, 0.98 | **0·010** |  |  |  |
|  | Team cohesion  (team) |  |  |  |  |  |  |  |  |  |
|  | NGO evaluation |  |  |  |  |  |  |  |  |  |
|  | **Personal** |  |  |  |  |  |  |  |  |  |
| Depression | Motivation | 1.05 | 0.94, 1.17 | 0·407 | 1.11 | 1.01, 1.21 | **0·038** | 1.05 | 0.95, 1.16 | 0·392 |
| Burnout EE | Motivation | 1.06 | 0.94, 1.20 | 0·372 | 1.11 | 1.01, 1.22 | **0·028** | 0.97 | 0.89, 1.07 | 0·587 |
| Burnout PA | Motivation | 0.92 | 0.86, 0.99 | **0·030** | 0.93 | 0.87, 0.99 | **0·045** | 0.95 | 0.88, 1.03 | 0·233 |
|  | Coping, problem solving |  |  |  |  |  |  |  |  |  |
| Depression | Coping, avoidance  Ref=Low-1.99 vs.  >2-highest | 3.32 | 1.33, 8.33 | **0·010** | 1.21 | 0.54, 2.71 | 0·636 | 1.68 | 0.73, 3.85 | 0·211 |
| Burnout PA | Coping, social support | 0.56 | 0.26, 1.21 | 0·142 | 0.44 | 0.21, 0.92 | **0·030** | 0.58 | 0.26, 1.31 | 0·192 |
| Depression | Health index | 0.67 | 0.46, 0.98 | **0·033** | 0.92 | 0.64, 1.31 | 0·630 | 0.74 | 0.55, 1.01 | **0·056** |
| Anxiety | Health index | 1.07 | 0.62, 1.83 | 0·819 | 0.78 | 0.54, 1.13 | 0·194 | 0.53 | 0.34, 0.84 | **0·006** |
| Burnout EE | Health index | 1.46 | 0.90, 2.36 | 0·141 | 0.84 | 0.59, 1.19 | 0·211 | 0.72 | 0.52, 0.99 | **0·035** |
|  | Alcohol use |  |  | 0·312 |  |  | 0·835 |  |  | 0·238 |
|  | Spirituality, fulfillment |  |  |  |  |  |  |  |  |  |
|  | Spirituality, maintaining |  |  |  |  |  |  |  |  |  |
|  | Spirituality, life purpose |  |  |  |  |  |  |  |  |  |
|  | **Exposure** |  |  |  |  |  |  |  |  |  |
| Depression | Child trauma | 1.70 | 1.00, 2.88 | **0·050** |  |  |  |  |  |  |
| Anxiety | Child trauma | 2.17 | 0.98, 4.82 | **0·057** |  |  |  |  |  |  |
|  | Family risk |  |  |  |  |  |  |  |  |  |
|  | Adult trauma |  |  |  |  |  |  |  |  |  |
| Depression | Extraordinary stressors | 1.71 | 1.28, 2.32 | **0·0003** |  |  |  |  |  |  |
| Anxiety | Extraordinary stressors | 1.65 | 1.06, 2.57 | **0·028** |  |  |  |  |  |  |
| Burnout DP | Extraordinary stressors | 1.38 | 0.99, 1.92 | **0·053** |  |  |  |  |  |  |
| Depression | Chronic stress |  |  |  | 1.12 | 1.03, 1.23 | **0·010** |  |  |  |
| Anxiety | Chronic stress |  |  |  | 1.12 | 1.01, 1.25 | **0·038** |  |  |  |
| Burnout EE | Chronic stress |  |  |  | 1.17 | 1.07, 1.28 | **0·001** |  |  |  |
| Burnout DP | Chronic stress |  |  |  | 1.17 | 1.05, 1.31 | **0·003** |  |  |  |
| Burnout DP | Trauma exposure category^†^ |  |  |  | 2.18 | 1.00, 4.75 | **0·050** |  |  |  |
|  | Recent stressful events |  |  |  |  |  |  |  |  |  |

EE = emotional exhaustion; DP = depersonalization; NGO = non-governmental organization; PA = personal accomplishment.

*All variables considered are listed in the table, but only those results are listed that had significant associations with any of the outcomes at approximately p <0·05 (in bold type).

^†^ Trauma exposures are defined as follows:

Category 1 = 0 trauma events.

Category 2 = 1–4 traumatic events.

Category 3 = ≥5 traumatic events.
