# Supplementary material for: Psychological Distress, Depression, Anxiety, and Burnout among International Humanitarian Aid Workers: A Longitudinal Study
Source: PLoS One. 2012 Sep 12;7(9):e44948. doi: 10.1371/journal.pone.0044948 (PMC3440316; doi:10.1371/journal.pone.0044948)
Supplement: Table S4 — Longitudinal multivariate generalized estimating equations model: demographic variables, exposure, organizational and other risk and mitigating factors affecting life satisfaction. (DOC) [file pone.0044948.s005.doc]

**Table S4: Longitudinal multivariate generalized estimating equations model: demographic variables, exposure, organizational and other risk and mitigating factors affecting life satisfaction**

| **Parameter** | **Adjusted**  **Mean Score** | **95% CI** | **p value** |
| --- | --- | --- | --- |
| **Time Period**  Pre  Post  Follow-up | 23.28  22.19  20.85 | 21.53-25.02  20.64-23.73  19.27-22.44 | **0.002** |
| **Sex**  Female  Male | 22.98  21.23 | 21.34-24.62  19.38-23.08 | 0·075 |
| **Marital status**  Not married  Married | 20.97  23.24 | 19.32-22.61  21.46-25.03 | **0·014** |
| **Job Function**  Non-manager  Head of mission | 20.99  23.22 | 19.49-22.49  21.31-25.12 | **0·014** |
| **Mental illness**  No  Yes | 23.01  21.21 | 21.62-24.40  19.11-23.30 | 0·082 |
| **Hardship assignment**  Yes  No | 21.83  22.38 | 19.98-23.67  20.82-23.94 | 0.539 |
| **Trauma exposure category*** |  | | |
| Traumatic stress category 1 | 21.64 | 19.75-23.51 | 0·589 |
| Traumatic stress category 2 | 22.63 | 21.12-24.12 |
| Traumatic stress category 3 | 22.04 | 19.10-24.98 |
| **Organization support** |  | | |
| Organizational support score 1 | 22.36 | 20.88-23.84 | 0·883 |
| Organizational support score 2 versus 1 | 22.08 | 20.53-23.63 |
| Organizational support Score 3 versus 1 | 21.89 | 19.58-24.17 |
| **Alcohol use**  Less alcohol use  More alcohol use | 23.12  21.09 | 21.48-24.76  19.53-22.65 | **0·003** |
| **Coping Avoidance Subscale**  A little  A lot | 22.85  21.36 | 21.37-24.34  19.70-23.02 | **0.015** |
| **Parameter** | **Regression Coefficient** | **Standard Error** | **p-value** |
| **Age** | -0.0221 | 0.0552 | 0.689 |
| **Chronic stress**  Sum | -0.0058 | 0.0921 | 0·950 |
| **Social support** | 0.1421 | 0.0611 | **0·021** |

AOR = adjusted odds ratio; CI = confidence interval.

Each variable in the table was adjusted for all other variables in the table. Time was also included as an adjustment variable in the analysis.

p values <0·05 were considered statistically significant and are in bold type.

* Trauma exposures are defined as follows:

Category 1 = 0 trauma events.

Category 2 = 1–4 traumatic events.

Category 3 = ≥5 traumatic events.
