# Supplementary material for: Psychological Distress, Depression, Anxiety, and Burnout among International Humanitarian Aid Workers: A Longitudinal Study
Source: PLoS One. 2012 Sep 12;7(9):e44948. doi: 10.1371/journal.pone.0044948 (PMC3440316; doi:10.1371/journal.pone.0044948)
Supplement: Table S5 — Job satisfaction versus risk/mitigating factors at post-deployment. (DOC) [file pone.0044948.s006.doc]

**Table S5:** Job satisfaction versus risk/mitigating factors at post-deployment

| **Parameter** | **Adjusted Mean Score** | **95% CI** | **p value** |
| --- | --- | --- | --- |
| **Sex**  Female  Male | 15.78  15.79 | 14.95-16.61  14.85-16.73 | 0.988 |
| **Marital status**  Not married  Married | 15.74  15.83 | 14.91-16.58  14.94-16.73 | 0·855 |
| **Job function**  Non-manager  Head of mission | 16.18  15.40 | 15.41-16.95  14.42-16.37 | 0·118 |
| **Hardship assignment** |  | | |
| Yes  No | 16.22  15.36 | 15.27-17.17  14.57-16.14 | 0·079 |
| **History mental illness**  No  Yes | 15.98  15.60 | 15.31-16.65  14.49-16.71 | 0·502 |
| **Traumatic stress exposure category*** |  | | |
| Traumatic stress category 1 | 16.15 | 15.18-17.12 | 0·576 |
| Traumatic stress category 2 | 15.61 | 14.90-16.33 |
| Traumatic stress category 3 | 15.60 | 14.04-17.16 |
| **Parameter** | **Regression Coefficient** | **Standard error** | **p value** |
| **Age** | 0.0130 | 0.0305 | 0.670 |
| **NGO evaluation**  Sum | 0.1415 | 0.0431 | **0·001** |
| **NGO work experience** Sum | 0.1100 | 0.0639 | 0·087 |
| **Team cohesion field leader** | 0.0651 | 0.0520 | 0·213 |
| **Team cohesion team** | 0.0696 | 0.0802 | 0·387 |
| **Chronic stress**  Sum | 0.0034 | 0.0505 | 0·946 |
| **Healthy habits index** | -0.1576 | 0.2175 | 0·470 |
| **Motivation**  Sum | 0.0110 | 0.0554 | 0·843 |

CI = confidence interval; NGO = non-government organization.

Each variable in the table was adjusted for all other variables in the table.

P values <0·05 were considered statistically significant and are in bold font·

* Trauma exposures are defined as follows:

Category 1 = 0 trauma events.

Category 2 = 1–4 traumatic events.

Category 3 = ≥5 traumatic events.

DP = depersonalization; EE = emotional exhaustion; NGO = non-governmental organization; PA = personal
